# Supplementary figures and images for: Supporting islet function in a PVDF membrane based macroencapsulation delivery device by solvent non-solvent casting using PVP
Source: PLoS One. 2025 Mar 12;20(3):e0298114. doi: 10.1371/journal.pone.0298114 (PMC11902058; doi:10.1371/journal.pone.0298114)

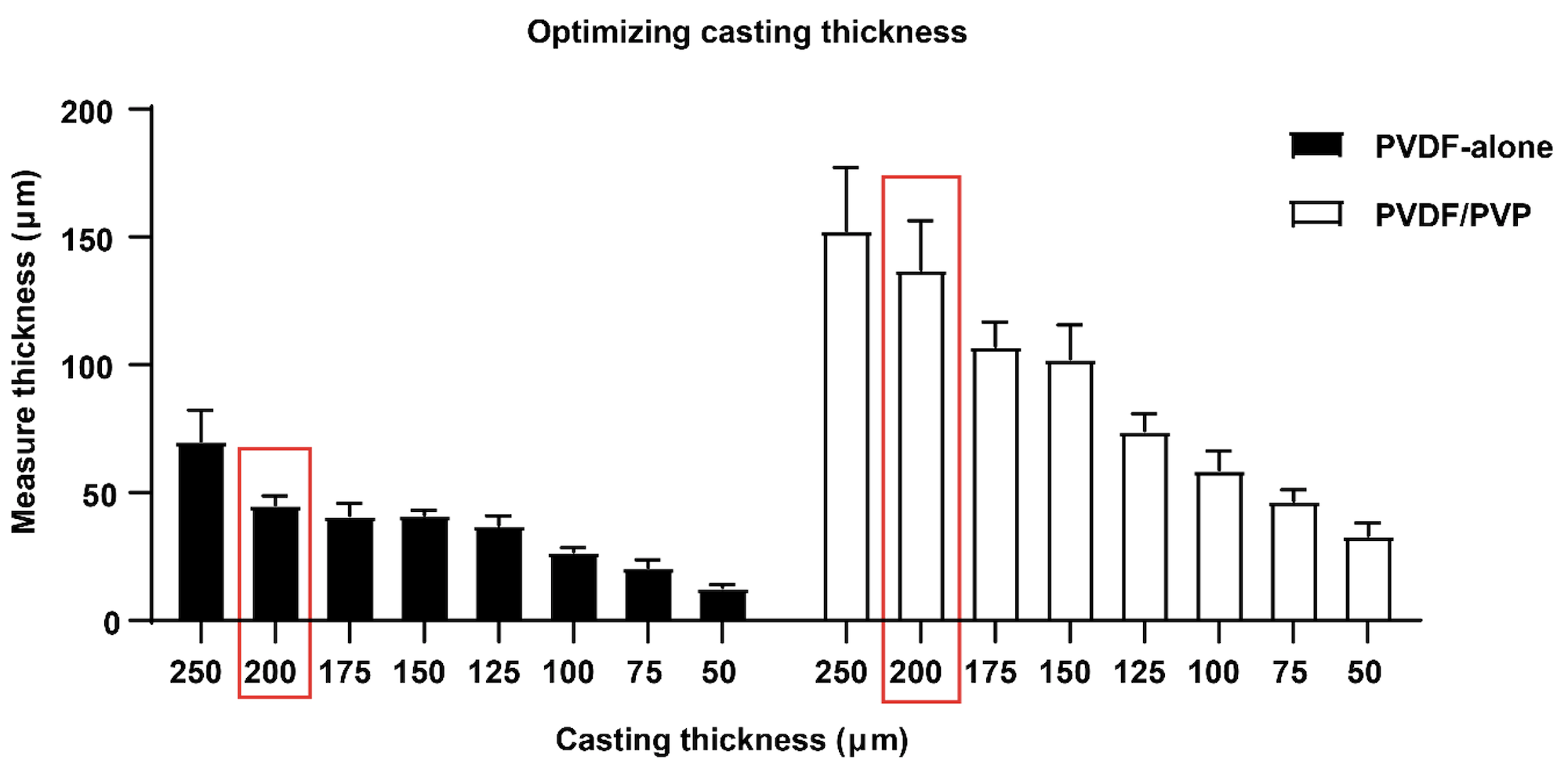

Supplement: S1 Fig — The red frame represents the selected casting thickness. (TIFF) [file pone.0298114.s001.tiff]

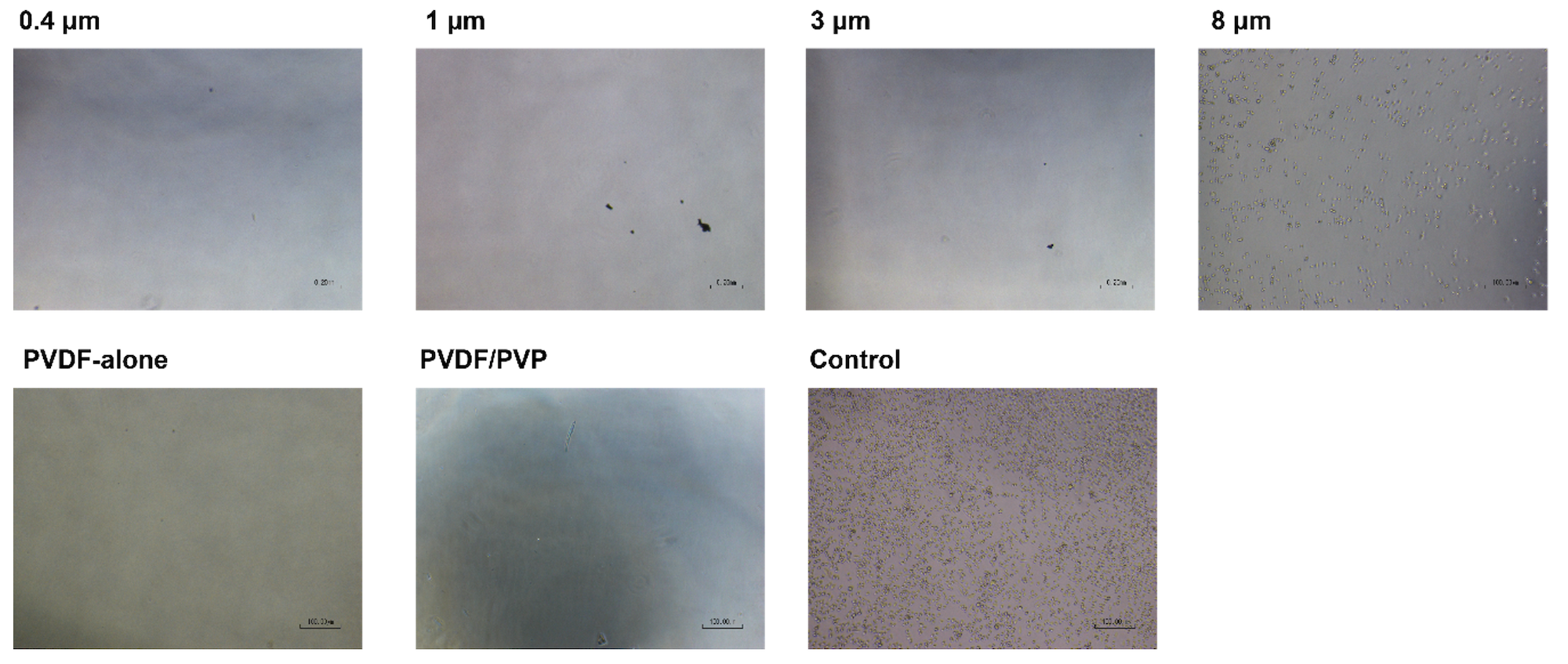

Supplement: S2 Fig — After 24h of culturing human primary macrophages in (modified) transwell inserts. Cells were detected in the control and 8 µm wells. (TIFF) [file pone.0298114.s002.tiff]

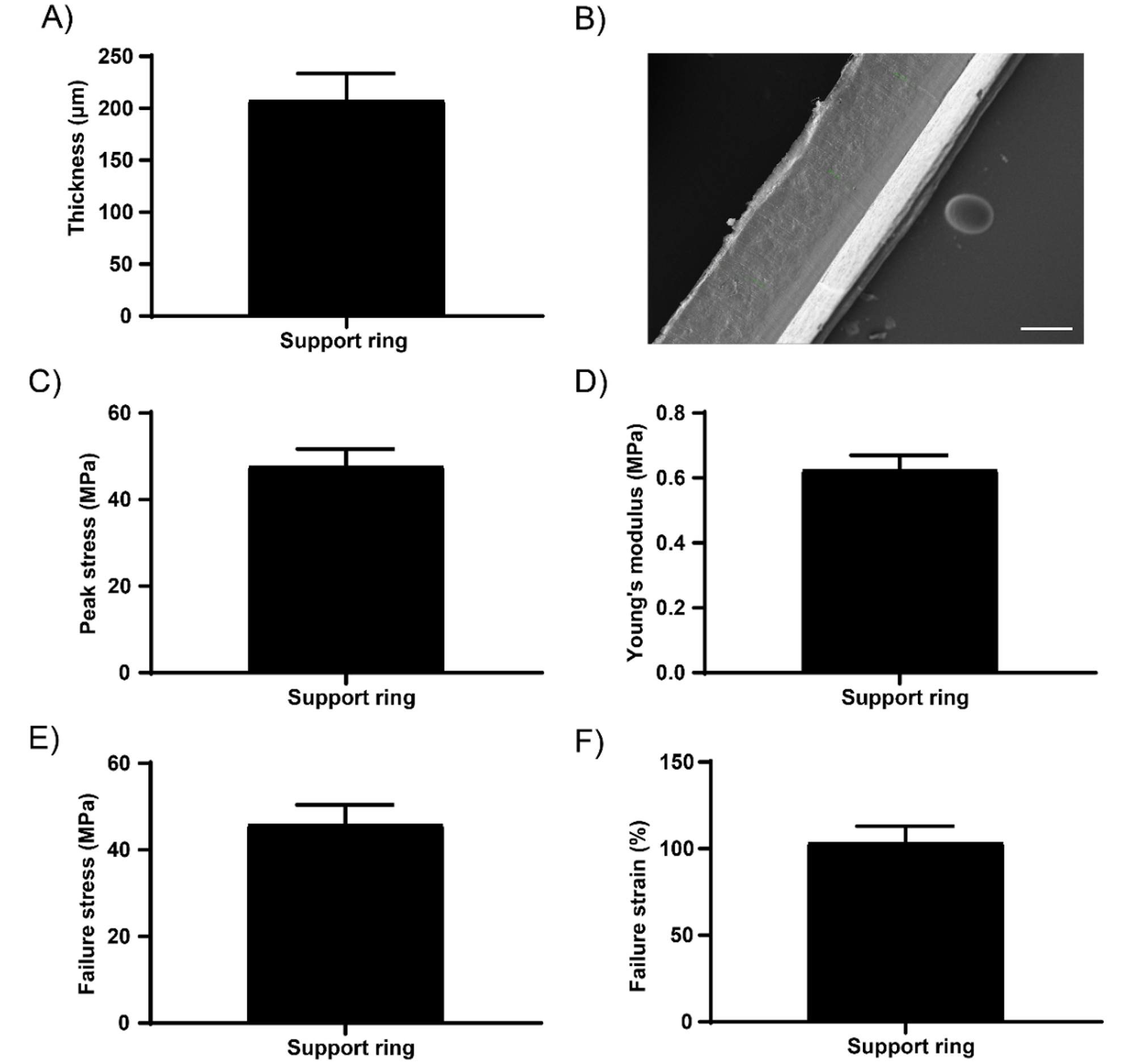

Supplement: S3 Fig — (A) Thickness of produced support ring measured from SEM cross-section images. Data presented as mean ± SD, n = 3. (B) SEM cross-section image of PVDF support ring. Scale bar represents 100 μm Mechanical properties of produced membranes (C-F). (C) Peak Stress, (D) Young’s modulus, (E) Failure Stress, (F) Failure strain. Data presented as mean ± SD, n = 5. Thickness 208 ± 25 µm, Peak stress 48 ± 4 MPa, Young’s’ modulus 0.62 ± 0.05 MPa, Failure stress 46 ± 5 MPa, Failure strain 103 ± 10%. (TIFF) [file pone.0298114.s003.tiff]

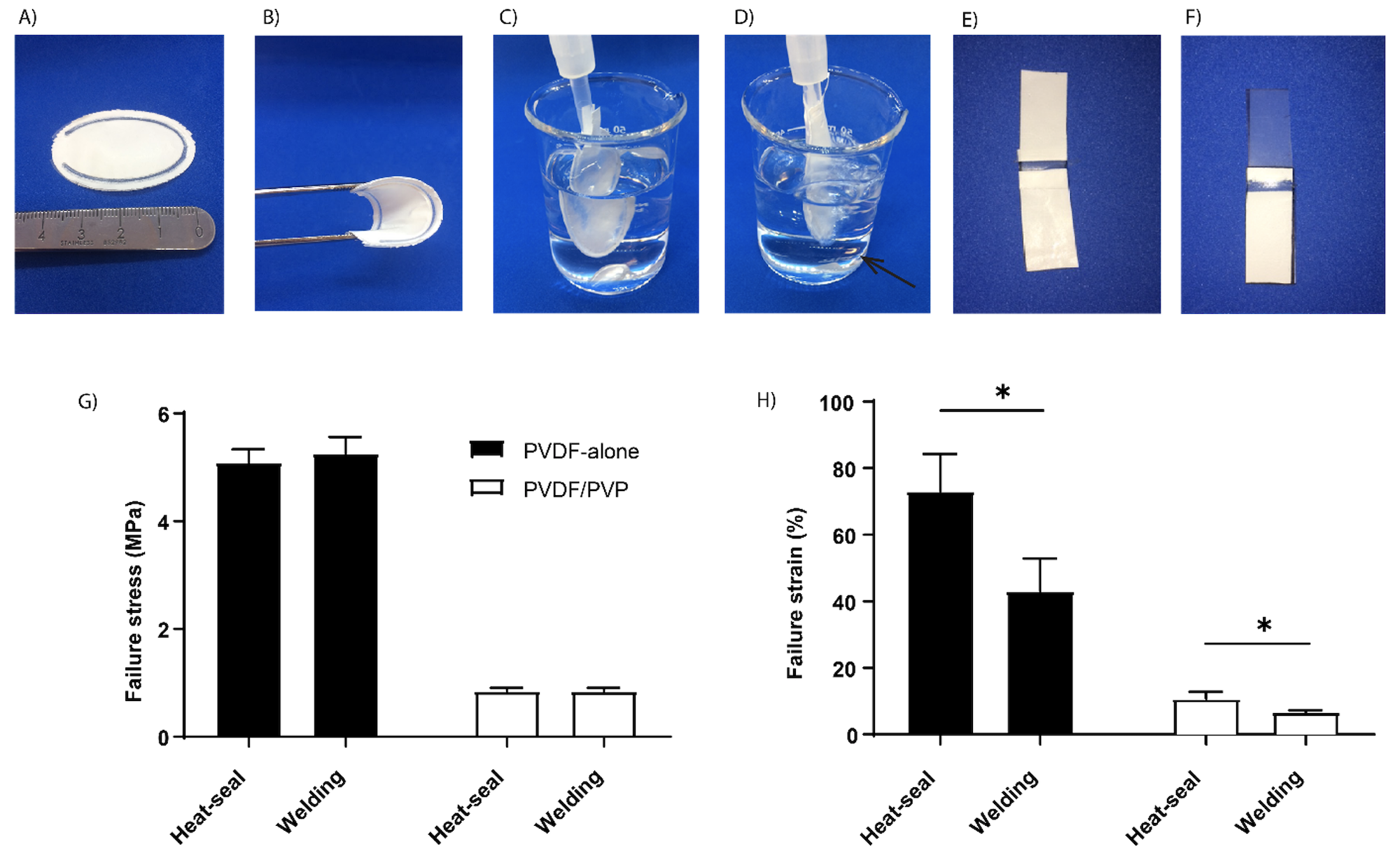

Supplement: S4 Fig — (A) Assembled encapsulation device. (B) Handling assembled encapsulation device. Camera shots of device testing for leakage (C-D). (C) Leakage free device and leaking device (D). (E) Heat sealing configuration with two membrane sheets. (F) Ultrasonic welding configuration with one membrane sheet and a support structure. Mechanical properties of heat and ultrasonic welded seals (G-H). (G) Failure stress and (H) failure strain of seals. Data presented as mean ± SD, n = 5. * P < 0.05, with student t-test. (TIFF) [file pone.0298114.s004.tiff]
