# Supplementary material for: Supporting islet function in a PVDF membrane based macroencapsulation delivery device by solvent non-solvent casting using PVP
Source: PLoS One. 2025 Mar 12;20(3):e0298114. doi: 10.1371/journal.pone.0298114 (PMC11902058; doi:10.1371/journal.pone.0298114)
Supplement: Attachment — Submitted filename: revision plos one.docx [file pone.0298114.s005.docx]

Dear author,

Thank you for submitting your manuscript “Supporting islet function in a PVDF membrane based immunoprotective cell delivery device by solvent non-solvent casting using PVP”.

I read with interest your paper and I believe that with some additional work it would be a valuable contribution to the literature.

However, I believe that in the current form, it is not suitable for publication.

The study presents the results of original research but experiments, statistics, and other analyses are not performed to a high technical standard, not described in sufficient detail and conclusions are not fully supported by the data presented.

While the mechanical characterization of the materials is sufficiently described, it is not clear the implication of having different pore sizes on different surfaces of the membranes. How does this affect the device assembly? Moreover, a lot of importance is given to the fact that the PVDF/PVP membranes allowed a greater diffusion of dextran, glucose, insulin and IgG compared to PVDF alone. Nevertheless, the fact that IgG diffuses through the membrane is an aspect that deserves more investigation. Even though the immunoprotection ensured by the size of the pores prevents the migration of macrophages (which is already known in the literature, as suggested by the author), it is not discussed whether this IgG diffusion could be detrimental to the islets' viability. Moreover, also “the permeability to inflammatory cytokines, such as IL-1β, histamine, TNF-α, and TGF-β” should be taken into consideration. Experiments in which these molecules are used during the culture of encapsulated islets and their effect on viability and function are likely to be included.

Similarly, co-culture systems with islets inside the device and macrophages outside could be helpful to confirm that they do not harm the islets.

In summary, I believe that further experiments on the biological validation of using this technology are necessary to complete this paper, ideally supported with results on their in vivo application.

I encourage the authors to resubmit the manuscript after performing new experiments to demonstrate the scientific validity of their technology.
