# Supplementary material for: Supporting islet function in a PVDF membrane based macroencapsulation delivery device by solvent non-solvent casting using PVP
Source: PLoS One. 2025 Mar 12;20(3):e0298114. doi: 10.1371/journal.pone.0298114 (PMC11902058; doi:10.1371/journal.pone.0298114)
Supplement: Attachment — Submitted filename: Plos one revison.docx [file pone.0298114.s006.docx]

Please use the space provided to explain your answers to the questions above. You may also include additional comments for the author, including concerns about dual publication, research ethics, or publication ethics. (Please upload your review as an attachment if it exceeds 20,000 characters).

Dear editorial office,

We have read the comments of all reviewers and made adjustments accordingly. The changes based on the reviewers’ comments and suggestions are addressed one by one and a description can be seen in blue in the following text. I have also indicated major changes by highlighting text in the manuscript in yellow to help discern the most important issues.

All the best,

Aart van Apeldoorn

Assistant professor

MERLN institute for technology-inspired regenerative medicine

Maastricht University

The Netherlands

Reviewer #1: In their manuscript ‘supporting islet function in a PVDF membrane based immunoprotective cell delivery device by solvent non-solvent casting using PVP’ the authors report on the construction of a PVDF microencapsulation device for islet cell transplantation. They enhanced permeability of the devices thanks to the incorporation of PVP while casting the membrane.

The manuscript is well-written and well prepared. The devices are well characterized by their mechanical and biological properties. Functional data are however less clear.

Moreover, a number of devices constructed with different biomaterials are under development or used for preclinical and clinical testing with islet cell tissue of different origin.It is unclear for the reader of the manuscript how this device can be used as a possible alternative while also focusing on the difficulties that are encountered during in vivo application of different devices for macro-encapsulation.

We thank the reviewer for the time and comments. We improved on the clarification and motivation and discussion about macro devices throughout the entire manuscript.

Some points may be addressed to clarify this:

The authors chose using PVDF/PVP as biomaterial for islet cell encapsulation because of lower oxidative stress to alpha and beta cells; however, strong data on biocompatibility, especially for the purpose of cell encapsulation are missing. Do the authors have data on the degree of pericapsular fibrotic overgrowth when using this type of device? It would be valuable to add this to the manuscript.

We highly appreciate the reviewer’s suggestion and we rewrote some parts of the introduction to clarify this better and have a clearer motive for the study for the readers to understand. We have done an extensive biocompatibility study done in small animals after this manuscript was written and submitted to PLOS. We choose not to include this study since placing both studies into one paper would also change the entire scope of this paper and prevent us from discussion both studies in depth. This manuscript focused on the design and fine tuning of membranes used for device manufacturing prior to extensive animal studies

How does the PVDF/PVP biomaterial differs from PTFE, which is used in most of the clinical applied devices in terms of mechanical, functional and biocompatible properties? Comparisons of diffusion properties and biomaterials with clinical used materials would be of added value to the manuscript.

We thank the reviewer for this question and we understand where this is coming from, since there is commercially device available for research (Theracyte™) composed of PTFE. It is hard to make a direct comparison between these biomaterials simply because they are different in nature and need to be processed in a completely different manner to create membranes from. Any comparison would likely show they are different but that is by definition the case. However, we addressed some of these differences in the introduction and added additional plus references to highlight this a bit better.

Islet cells need to respond quickly to increase in blood glucose levels; this might be a disadvantage of the devices since diffusion of glucose (equilibrium after 3h)and insulin (equilibrium after 24 hours) seems rather slow. Can the authors comment on this?

This is indeed a very relevant comment, and a topic continuously discussed in the T1D field. We have elaborated on this based on the reviewer’s advice which is added to the discussion.

The diffusion setup is based on diffusion of molecules between a high concentration compartment and a no concentration compartment, the driving force of diffusion in this setup is related to the difference between concentrations on either side. The diffusion rate therefore slows down during the experiment until it reaches zero, one can imagine a different mechanism occurring in vivo where the insulin or glucose is continuously removed the driving force is expected to stay high.

It is unclear how this slow diffusion affects the real glucose stimulation index: the comparison between the free islet cells with the encapsulated ones is difficult to make since number of IEQ and media concentrations seem different; it would be interesting to know basal and stimulated insulin synthesis per IEQ or beta cell. Were experiments of different conditions performed in parallel with tissue of the same donor? How was the number of IEQ determined?

We thank the reviewer for the comments, these are multiple questions we’ll address here one by one. We chose to measure insulin secretion compared to total insulin present in all beta cells in each condition. One can assume that beta cells in general contain more or less a similar amount of insulin granules, thus insulin. This relative measurement compensates for any differences between number of IEQs used per experiment like one would use DNA or IEQ. This is in line with literature in the field see for example: Jean-Claude Henquin, Glucose-induced insulin secretion in isolated human islets: Does it truly reflect β-cell function in vivo?, Molecular Metabolism, Volume 48, 2021, 101212, <https://doi.org/10.1016/j.molmet.2021.101212>. In which total insulin is used

One islet equivalent (IEQ) is an islet of diameter 150 μm, typically containing 1,500-2,000 cells, including 40-60% beta cells. Islets of different sizes are volumetrically adjusted to IEQs for each experiment

Yes, experiments of the different conditions where done in parallel with donor tissue derived from the same donor during each experiment, otherwise donor to donor variation would overshadow any of the outcomes. In the end outcomes with the different donors where averaged and depicted in the relevant graphs as such. We have added some additional explanation in the caption of the graphs to clarify this

Adding perifusion experiments with devices and free cells would be of interest.

We agree with the reviewer that this could be an interesting addition. However, afterwards repeating this would mean new human donor islets would have to be required, a minimal of 3 is needed while human donor islets are scarcely available, and all assays for all the other *in vitro* analyses would also have to be repeated. We have used GSIS comparable to others publications in the field to show that insulin comes out in similar quantities as we see for free floating non encapsulated cells. This is why a standard GSIS has been used similar to previous publications on this topic. A perfusion assay could indeed be used to do a in line time-based study, however one has to realize that these dynamics likely completely change in vivo.

Macro-encapsulation devices with islet cells from different sources (allo, xeno, iso, stem cell generated) have been shown to restore metabolic function in immune deficient rodent models; this is necessary to confirm possible in vivo islet cell function within the devices.

Are in vivo data available?

Unfortunately, not yet, since this paper is about the design, engineering and characterization of these new PVDF based macroencapsulation devices, describing a new design, using a specific membrane fabrication technique to create porous membranes from which a macroencapsulation device can be made. We focused thus primarily on the characterization and fabrication of the device rather than *in vivo* studies to make sure we understand the working principle prior to elaborated in vivo studies. We have some preliminary data with human islets in a non-diabetic mouse, showing the presence of human c-peptide after implantation. However, the number of animals used in this pilot study does not warrant enough power for a proper statistical analysis and can only be considered as anecdotal observations for now. A more elaborate in vivo study based on this paper will be a next step. We have been working towards a long-term study in a rat model. However, these studies are still ongoing and would significantly change the focus of this manuscript away from the design, engineering characterization of these devices.

Some minor remarks and questions:

The authors use islets at 75% purity; how was this determined?

As described in the material and methods, human donor islets were provided by our colleagues from the Leiden University medical center’s clinical islet isolation and transplantation center. Islet purity is commonly determined in the field by a standard DTZ staining and accompanying microscopic evaluation. DTZ stains zinc associated to insulin red, which can be used to distinguish islets from non-endocrine tissue in isolation preps using light microscopy. This is a commonly used and widely accepted method in islet isolation to determine purity of islet preps in clinical islet isolation and transplantation centers, in research and clinical applications. We didn’t feel therefor that it’s necessary to describe the determination of purity in detail since this is standard practice for releasing islets. We’ve added an additional sentence to the M&M to clarify this further. This generally accepted method in the field of clinical islet transplantation is well-described in literature see for example:

Pisania A, Weir GC, O'Neil JJ, Omer A, Tchipashvili V, Lei J, Colton CK, Bonner-Weir S. Quantitative analysis of cell composition and purity of human pancreatic islet preparations. Lab Invest. 2010 Nov;90(11):1661-75. doi: 10.1038/labinvest.2010.124. Epub 2010 Aug 9. PMID: 20697378; PMCID: PMC2966538, and James Lyon, Aliya F Spigelman, Jocelyn E Manning Fox, Patrick E Macdonald 2021. Human Islet Quantification and Purity Assessment. **protocols.io** [https://dx.doi.org/10.17504/protocols.io.bus3nwgn](https://dx.doi.org/10.17504/protocols.io.bus3nwgn)

The authors examine the immune-protective capacity of the membranes by evaluating the degree of macrophage migration; this is however only a portion of immune-reactivity. Could the authors comment on this?

We have added a revised paragraph on cytokines and IgG in which we have changed the wording related to blocking the entry of macrophages into the device to clarify more about this topic.

How was the pore diameter calculated?

We apologize for not describing this clearly in the M&M. We have now added a description in the M&M to clarify this further. Pore diameter was determined by SEM measurements on the surface of the membranes. For example, in figures 1 and 4 the pores can be clearly seen on the surface of all samples used. One can use the measurement tool in the operating software of the scanning electron microscopy to determine dimensions accurately by taking several randomized measurements on pores.

In the introduction the authors refer to one of their previous studies using directly vascularized polymer-based cell delivery devices transplanted in the fat pad; however, the correct reference is missing in the manuscript.

We have now inserted a reference for this omission.

How many cells could be loaded maximally in a device without causing cell death? One restriction of the use of macrodevices is the number of cell material that can be loaded inside the device. How many islet cells can be injected in the devices proposed in the manuscript? At what cell density could the cells survive and function in vivo?

We thank the reviewer for these interesting and very relevant questions. At this moment we can not say for sure. We agree with the reviewer this could be an interesting study and should be done in general for all macrodevices.

This would require an extensive study on oxygen distribution in macroencapsulation devices, perhaps supported with computational modelling, versus empirical *in vitro* studies on islet function and cell survival. If one wants to do a complete study, it should include probably beta cells in suspension, pseudoislets, stem cell derived islets and primary islets from multiple human donors and perhaps even different animal species to provide a precise answer. In addition, such a study should maybe also include a head-to-head comparison with other macrodevices.

The maximum volume which can be injected into these rat sized devices in our paper is about 1.5 ml. Based on modelling studies of others and our own, islets with a diameter of 150 micrometer are optimal in terms of minimizing hypoxia, while the distance between these islets can still be quite small ~40 micrometers before detrimental low oxygen levels are reached. However, competition in closed macroencapsulation devices for the necessary nutrients limited by diffusion rates can induce a significant amount of cell death.

Stem cell derived beta cells are generally thought to cope better with suboptimal oxygen levels due to their immaturity compared to primary terminally differentiated beta cells, which might allow for higher cell concentrations. Apart from these *in vitro* studies extensive implantation studies would be warranted. The location of implantation might have a strong effect on cell survival, while lack of vascularization in certain sites exacerbates the lack of oxygen supply to densely packed encapsulated cells leading to more cell death.

If needed we can add the above discussion to the discussion paragraph provided it doesn’t make the manuscript too long.

Reviewer #2: Dear author,

Thank you for submitting your manuscript “Supporting islet function in a PVDF membrane based immunoprotective cell delivery device by solvent non-solvent casting using PVP”.

I read with interest your paper and I believe that with some additional work it would be a valuable contribution to the literature. However, I believe that in the current form, it is not suitable for publication.

The study presents the results of original research but experiments, statistics, and other analyses are not performed to a high technical standard, not described in sufficient detail and conclusions are not fully supported by the data presented. While the mechanical characterization of the materials is sufficiently described, it is not clear the implication of having different pore sizes on different surfaces of the membranes. How does this affect the device assembly?

We thank the reviewer for the positive comments and we agree that more detail is needed in the materials and methods section to fully appreciate the data for the general reader. Based on the previous comments from the other reviewer we have now elaborated on some of the methods used, and also added a more extensive in-depth discussion on the outcomes in the discussion section of the manuscript

Device assembly and pore sizes: Interesting question, but the increased pore sizes as a result of adding PVP doesn’t affect the device assembly at all, the pore sizes are in the submicrometer range and can hardly be seen by the naked eye and therefore have no significant influence on the device assembly. The membranes can be used and handled like a dense film for device assembly.

Moreover, a lot of importance is given to the fact that the PVDF/PVP membranes allowed a greater diffusion of dextran, glucose, insulin and IgG compared to PVDF alone. Nevertheless, the fact that IgG diffuses through the membrane is an aspect that deserves more investigation. Even though the immunoprotection ensured by the size of the pores prevents the migration of macrophages (which is already known in the literature, as suggested by the author), it is not discussed whether this IgG diffusion could be detrimental to the islets' viability. Additionally, also “the permeability to inflammatory cytokines, such as IL-1β, histamine, TNF-α, and TGF-β” should be taken into consideration. Experiments in which these molecules are used during the culture of encapsulated islets and their effect on viability and function are likely to be included.

Similarly, co-culture systems with islets inside the device and macrophages outside could be helpful to confirm that they do not harm the islets. In summary, I believe that further experiments on the biological validation of using this technology are necessary to complete this paper, ideally supported with results on their in vivo application. I encourage the authors to resubmit the manuscript after performing new experiments to demonstrate the scientific validity of their technology.

We thank the reviewer of these comments and realize these are somewhat similar to comments from the previous reviewer. To make sure we address the protective properties in an appropriate manner we have made changes in the text throughout the entire manuscript to highlight the fact that we are trying to find a sensible compromise between insulin, glucose and nutrient diffusion to and from the encapsulated cells on the inside of the closed device, while preventing direct cell-to-cell contact with cells at the outside. We are aware of the effects of cytokines, and to a lesser extend IgG. However, by blocking these molecules it would also mean a significant reduction in diffusion of the endocrine hormones a problem all immunoprotective islet delivery macrodevices suffer from. We choose to find a compromise and not significantly hinder islet function and survival instead. We have made extensive textual adjustments in the manuscript to clarify this better and also make the proper claims about the protective nature of the device.

Reviewer #3: The manuscript titled: ‘’Supporting islet function in a PVDF membrane based immunoprotective cell delivery device by solvent non-solvent casting using PVP’’ by Denise F.A. de Bont et al., focuses on the optimization of membrane fabrication PVDF-PVP ratio with respect to the diffusion properties of the device that can be tailored to support human islet function while preventing at the same time the membrane entry of primary human macrophages that damaged beta cells.

Even if the study presented the findings of original research focusing on new therapeutic approaches for Type 1 diabetic (T1D) patients who are lifelong dependent on insulin therapy to keep their blood glucose levels under control, the experiments, particularly the in vitro experiment, and other analyses were not performed to a high technical standard and were not presented and described in sufficient detail. Consequently, the conclusions are not supported by the appropriate data. A large quantity of data must be added to support the authors' hypothesis, which now is missing from the text.

We thank the reviewer for the remarks and realize we have maybe overstated some issues since we are not actually testing these devices in a clinical setting yet. We have made textual changes throughout the entire document (introduction, materials and methods, and discussion) to clarify the goals of this study better. In addition, we have made considerable textual changes to better describe the methods used and elaborate significantly on the discussion of the outcomes of this study in the discussion section.

Major points:

-There is a need of a test that validates the presence of pure islets after culture on the device (e.g., the Dithizone assay), as well as an evaluation of their functioning throughout time in culture and morphologic characterization. I suggest the gene expression characterization for the islets that are cultured in PVDF-PVP device at short and long time in culture.

We are not entirely sure what the reviewer means with pure islets. For each experiment we used human donor islets with an average purity of 75%, we compensated for this and made sure in each device, and for each donor, a similar amount of IEQ was seeded into each device (see description in the material and methods) Human donor islets can only be kept in culture for a week until they start deteriorating and show central core necrosis. We chose to compare function of free-floating islets and encapsulated islets at day 8 since we expect to see major differences at said time point, while at the day of seeding no differences will be found for obvious reasons. Our functional data show that no major differences can be found at day 8 of culture, it is thus reasonable to assume that prior to this time point no major differences have occurred since islet function is the same between the two conditions at day 8. Live/dead assays corroborate this as well. We agree that gene expression analysis is indeed an interesting additional measurement but will not likely show any significant differences therefor while islet function remains the most important readout.

-In the manuscript are used INS-1E rat insulinoma cells, machophages and human islets. While the function of using macrophages is obvious, it is not clear why, in the last paragraph, human islet cells are used in place of the INS-1E rat insulinoma cells that were previously studied for cytotoxicity of membranes. In the paragraph and the discussion section, I suggest a more thorough explanation of the experimental strategy.

We thank the reviewer for this comment and we realize this indeed can be better described in the material and methods section, and thus changed the description accordingly. INS1E insulinoma cells are used as a model, and proof-of-concept cell line. We chose to use these cells to test for cytotoxicity in a more standardized manner since human donor islets have a heterogenous cell composition, they contain 5 endocrine cell types and additional supporting cells (endothelial, fibroblasts etc.) and are highly subject to donor to donor variation. We performed a cytotoxicity study similar to what is usually done with fibroblasts for biomaterials testing described in the ISO 10993-5: MTT & MEM Elution Test. This test involves cell counting after exposure to an extract from a biomaterial or device. If one would do this with islets, one would have to dissociate islets into individual cells using enzymatic digestion, which in itself leads to cell death and is usually very inefficient. This is why INS1E cells are a superior standardized model for cytotoxicity testing.

-The goal of the manuscript is preventing the entry of primary human macrophages that damage beta cells with the use of this device, but in the paragraph ‘’diffusion of small molecules through membranes’’ the authors demonstrated the capacity of IgG to pass through the membrane. This event could damage the efficacy of the human islet functionality? I suggest discussing this point and include new experiments that verified this point.

Similar to the above-mentioned reviewers comments on this, we have now addressed this issue by including an elaborated and clearer discussion on this topic to ensure we are not overstating the protective nature of the device, but also addressing the major challenge in engineering macroencapsulation devices for islets and beta cells.

-Moreover, I suggest reviewing the material and method section. Experiments are not described in an appropriate manner. in particular in the MTT assay paragraph, what do you mean for extraction samples? Are they the device with the different cells or the cells are removed from the device and then plated alone on the 96 well in order to obtain 50% of the confluent monolayer? Consider explaining the experiments more carefully.

Similar to the comments from the previous reviewers. We have now changed the wording in the materials and methods and discussion sections at multiple locations to clarify this better and provide a better explanation of the experimental setup and outcomes we hope this solves this issue. In addition, we did both an additional spell and grammar checks to make sure this is described in the right manner.

Minor points:

-In the manuscript are present some mistakes (e.g. line 172). I request a spell-check of the text.

We apologize for this omission and similar to the previous comment We have done an additional spell and grammar check on the entire manuscript and have rewritten some sentences accordingly to provide for improved reading.

Reviewer #4: Paper entitle “Supporting islet function in a PVDF membrane based immunoprotective cell delivery device by solvent non-solvent casting using PVP” PONE-D-22-17075 appears to be carefully and scientifically conducted with regard to the fabrication and characterization of the proposed new material the PVDF/PVP. Unfortunately, the whole part concerning the interaction of the device with islets or cells for the purpose to demonstrating its safety and immunoprotective capabilities does not appear to be appropriate either as a methodology or as an interpretation of the results. The data presented are in no way sufficient to define the device as immunoprotective. The only data Authors show is that passively macrophages (moreover, cultured under conditions that do not adhere to the vendor's recommendations) do not pass through the pores created on the PVDF/PVP.

We thank the reviewer for the comments and advice. As was mentioned before we have now made considerable textual changes not to overstate the protective properties of the device. In relation to the macrophage experiments, the control samples in our tissue culture experiments clearly show that the primary macrophages can migrate through pores when their size permits in our experiments. There is thus no need to include any additional chemoattractant or otherwise to stimulate migration of macrophages. In addition we have added an elaborate discussion on diffusion of

Confusion is also made between human islets and beta cells: it is one thing to use whole islets taken from cadavers and another to use beta cells purified from them.

We apologize for the confusion, beta cells (or the insulinoma cells), and the pseudoislets made from them (a method also published by others than ourselves) used in this study, serve as a model for islets. In order to test the device properly after these proof-of-concept studies one needs to use primary islets. In our study we haven’t isolated beta cells from primary islets, which wouldn’t make sense anyway since clinical islet transplantation and comparable studies have never been done with primary beta cells purified (isolated) from islets. Also, in light of the developments in the past 6 years with stem cell derived beta cells, the pseudoislets used in this study can also serve as a model for these.

The work should be thoroughly reviewed and presented as a characterization of the material and first circumstantial in vitro evaluation of the safety of the device.

We have now made considerable textual changes throughout the entire manuscript to clarify this better, the manuscript is exactly written for the purpose of characterization of a proof-of-concept design of the macrodevice while using *in vitro* models and finally primary human donor islets to show cell behavior in these closed devices.

To demonstrate the immunoprotective capabilities of a new material or device many other evaluations must be added (e.g., in vitro co-culture device empty or with islets and PBMCs from donors, followed by lymphocyte typing with cytokine and chemokine assay, macrophage migration assays with device containing chemoattractant first and then human islets, transplantation into animal models first immunocompromised for assessment of safety and foreign body reaction in vivo and then immunocompetent of the empty device and then containing islets, lymphomonocyte caracterization of transplanted animals, cytokine and chemokine assays under the various conditions, etc... ).

The discussion is by no means exhaustive, some concepts are already said in the introduction and therefore do not need to be recalled in this section. In several places it appears confusing because it is difficult to understand whether the authors are referring to their own data or to the literature, and often comparisons with devices presented by other groups do not seem appropriate. Also, the issue of where to transplant the device and the number/surface of devices needed for the correction of diabetic hyperglycemia are not addressed, it would be useful to address this issue to fully understand the potential of the proposed device.

The various critical issues are detailed below:

Major revisions

Title “Supporting islet function in a 1 PVDF membrane based immunoprotective cell delivery device by solvent non-solvent casting using PVP” It is not appropriate to talk about immunoprotection based on the data provided; the title should be changed.

Based on the reviewer’s comments we have changed the title accordingly to: A PVDF membrane based closed cell delivery device by solvent non-solvent casting using PVP

Lines 39-41 “We report on the creation of porous polyvinylidene fluoride (PVDF) membrane-based devices for islet and beta-cell transplantation.” In the paper, the authors do not test beta cell transplantation in any way; these should be removed from this and other sentences.

Based on the reviewer’s comments we changed this sentence into: “We report on the characterization and creation of porous polyvinylidene fluoride (PVDF) membrane-based devices intended for islet and beta-cell transplantation.” Better describing the intended goal of the paper.

Lines 45-47 “We showed that the use of a water-soluble pore forming reagent, PVP, can significantly increase glucose diffusion through these membranes without compromising their immunoprotective properties.” The immunoprotective properties of the device are not demonstrated by the data presented and therefore the sentence should be changed.

Based on the reviewer’s comments, changes were made accordingly, we showed that the use of a water-soluble pore forming reagent, PVP, can significantly increase glucose diffusion through these membranes without compromising their cell protective properties.

Lines 55-56 “….the membrane can still serve as an immunoprotective barrier preventing the entry of primary human macrophages and damaging beta cells Immunoprotection is quite a different concept and must be defined by very specific methods . Macrophages may remain outside but there is nothing to prevent them from being activated and secreting cytokines and chemokines .

We agree with the reviewer’s advice on rephrasing this and we also included a more elaborate discussion on the protective nature of the device. We’ve made specific textual changes accordingly: the membrane can still serve as a protective barrier preventing the entry of primary human macrophages and damaging beta cells

Lines 96-97 “Here we report on the development of an immunoprotective extrahepatic islet delivery device....” It is not correct to talk about “extrahepatic” sites instead alternative implantation sites on which to graft the device should be clearly defined.

Macrodevices are per definition applied extrahepatically. In clinical islet transplantation the standard is to infuse the islets via the portal vein into the liver. Macrodevices will always be implanted outside the liver due to their physical dimensions ergo “in an extrahepatic site” Although many implantation sites have been suggested and also tried in the field there is yet no consensus on what the most optimal implantation site for these devices is. The term extrahepatic is thus used in line with discussions in the field, indicating that islets or beta cells are not transplanted in the liver’s vasculature like is done in CIT.

Line 156 Explain the rationale for the use of INS-1E cells. If they are used as a control of whole insulae, they are not very comparable because they represent tumor cells derived from beta cells only.

The reviewer is correct in stating that these cells are not “true” beta cells. However, they have been used in the field by many others for conceptual studies and as a model for beta cells. There is an explanation in the text which states that these cells serve as a model, prior to the follow up studies in this paper with human donor islets.

Line 232- “The donor chamber was filled with a PBS solution containing 2 mg/mL 3-5 kDa FITC-dextran (Sigma-Aldrich, the Netherlands), 20 mM D-glucose solution …… “ The method described is not clear it seems that everything is put together at the same time in the same donor chamber. Rewrite the sentence.

We have rewritten this sentence and also did an additional grammar check on this paragraph to clarify this better.

Line 266 and figures 4K) 4L). “….that is free-floating control…” What is meant by this control? In all other analyses, PVDF alone was compared with PVDF/PVP. Please explain further and correct.

Each condition, free-floating controls (islets cultured on a non-adherend tissue culture plate a method used commonly in the field), PVDF-alone and PVDF/PVP devices, were tested three times independently. Free-floating controls are used in the field as a measure of how the basic islet function in human donor islets looks like without them being influenced by experimental conditions such as the presence of a biomaterial or other experimental conditions. This is needed since there is a large variation between islets obtained from individual donors not only functionally, but also purity and IEQ can vary. These controls show how beta cell function in islets is without any other influence.

Line 269 “Immunoprotective capacity of membranes” The title of the paragraph is inappropriate. From what is reported, the test is carried out using a device that does not contain any kind of biological material, so what is being tested is solely the ability of macrophages to react to the presence of the membrane. A more effective motility and invasiveness test would require the presence in the device of cells or molecules that can specifically attract macrophages toward a chemo-attractant gradient. In addition The 10% serum itself is used as a chemo-attractant for macrophages. So why are macrophages not cultured in serum free medium for this test?

The controls used in this experiment (membranes with larger pore sizes) show clearly that macrophages can migrate through these membranes, while this is not happening in the tailor made PVDF or PVDF/PVP membranes, regardless of the presence of serum or a specific chemo attractant. Use of a chemoattractant is thus not necessary to show the cell blocking nature of the membranes

Furthermore, in the paragraph on culturing macrophages (lines165-166) it is stated that they are cultured in "in Dulbecco's Modified Eagle Medium (DMEM) high glucose supplemented with 10% (v/v) FBS"....but the specifications of the company from which they are purchased recommend using pre-coated flasks with “Human Macrophage Primary Cell Culture Complete Extra-cellular Matrix”. Please explain the reasons for this type of culture.

We understand the comments of the reviewer. The flasks recommended and sold by the supplier are precoated with ECM (of unknown composition) to support cell proliferation of macrophages. However, precoating the tailormade membranes with ECM introduces an unknown factor, apart from the fact that one doesn’t know the exact composition of the commercially sold ECM coating. Moreover, PVDF is a rather hydrophobic biomaterial which prevents adsorption of water-soluble biomolecules to its surface. The SEM data clearly show that macrophages are adhering to the surface of the membranes, since we were interested in migration rather than proliferation and did not want to introduce an unknown factor, such as Human Macrophage Primary Cell Culture Complete Extra-cellular Matrix, of which the composition is no disclosed by the supplier, since the outcomes of the comparative study clearly show this is not needed we haven’t used a precoating step of membranes in our study.

Line 285 Why do the authors use different islets numbers between the device and the control ? When comparing results, even calculating them as a stimulation index could lead to errors. It would be a good idea to use the same IEQ number or explain the reasons for the choice made.

Free floating controls are typically done in non adherend tissue culture well plates. These tests are done to ensure that islet function in an experimental condition (in this case a device) can be compared to an untreated condition (free floating). We and others have published on many occasions using these controls in the past, which is generally accepted in the field as a proper functional test for islet function. Stimulation indices are always calculated based on insulin secretion in the first low glucose concentration and high glucose concentration determined by the GSIS test. In order to compensate for the number of IEQs between conditions and donors we used relative insulin secretion related to total insulin present in both the control and experimental condition to account for the IEQ difference. One can either use total insulin, DNA or ATP etc. to compensate for this difference.

Line 294 Viability should also be done at time 0, when the device is refilled, for a more appropriate comparison At the end of the experiment how many islets are recovered of the 3,000 IEQs originally put in?

We are not sure what the reviewer means with refilled, the devices are seeded at time 0 and then kept in culture for a week at which time point a comparison is made between free floating controls and islets in the device. None of the devices is refilled at any given time point. At time 0 one doesn’t see any differences since the islets haven’t yet been exposed to experimental conditions at this time, which occur over time i.e. after 7 days. At the end of each experiment total insulin is measured of all islets in free floating conditions and devices which requires lysis of all cells so no islets will be left after this destructive assay.

Line 379 Cytotoxicity should also be assessed on whole islets not only on INS.

We respectfully disagree with the reviewer for the following reasons. We have performed the cytotoxicity test according to ISO10993-5 which requires a monoculture of cells, not 3D cell aggregates with a heterogenous composition. The ISO10993-5 is a standardized protocol used for testing cytotoxicity of medical devices and biomaterials. Cytotoxicity testing is done by looking at proliferation and cell number during cell culture when exposing cells to an extract made from a device or biomaterial in comparison to a negative control. Primary islet cells do not proliferate since they’re terminally differentiated and cell counting in 3D cell aggregates is not accurately possible, while dissociation of islets would lead to cell death and is a very inefficient technique. Any outcomes of cytotoxicity tests on whole islets would lead to unclear outcomes and it doesn’t adhere to the iso standard for cytotox tests.

Line 382 Figure 3A not 4A

Line 385 Figure 3B not 4B

We’ve made corrections accordingly

Line 418 “FITC-labelled insulin reached equilibrium after 24 h in PVDF/PVP membranes as 47 ± 2% of insulin was diffused, compared to whereas 28 ± 5% was diffused in PVDF-alone membranes at the same time point (Fig. 4G).” Twenty-four hours is a really long time to reach equilibrium in PVDF/PVP membranes especially if you want to consider implanting this device in vivo. The islets inserted in the device are able to sense in real time the glucose present in the culture medium but the insulin they release is not able to diffuse from the device as fast, this could compromise metabolic control. The graph of static incubation presented as an index is not sufficient to understand whether the proportion of insulin produced is proportional to the glucose administered, it only shows that islets respond to the increase in glucose concentration even in the device. It should be discussed appropriately in discussion.

This is a fair comment and is similar to the previous reviewer’s comment. We have added a substantial paragraph elaborating on this issue in the discussion

Line 446 “ Immunoprotective function of PVDF membranes” Title is inappropriate, with the illustrated experiment only the ability of macrophages to spontaneously pass through holes is determined but not the immunoprotective character of the device.

We have changed the title accordingly

Line 503 “Immunoprotective beta-cell delivery devices…” the Device is tested only on whole islets from donor cadaver there is no data on purified beta-cells correct or enter corresponding data. The word “Immunoprotective” is not used correctly and should be changed.

We understand the confusion about the wording of the term. Beta cell delivery devices, beta cell replacement devices, or islet delivery devices is terminology used interchangeably in the field of type 1 diabetes and islet transplantation for the same type of device.

The reason for this is that primary islets or islets made from stem cell derived beta cells have a similar 3D appearance and devices made for islets are also made with the intention to use them with stem cell derived beta cells. It is not common practice to use beta cells as a single cell suspension or use primary beta cells isolated from islets. The latter is a very inefficient wat to obtain a sufficient amount of beta cells. We are using human donor islets in this study and not purified beta cells from donor islets.

We checked the manuscript for consistent use of the wording for the device.

“Discussion” : Some concepts are already said in the introduction and therefore need not be recalled in this section (line 517 for example). It needs to be rewritten considering previous recommendations. Also, the possible implantation site should be discussed prospectively, in number of devices to be used based on the number of IEQs it contains. The device presented is an oval of 44X28 mm, containing 3000 IEQs. To achieve blood glucose correction in an 80 kg diabetic man, it is estimated that at least 1000000 IEQs are needed...so could the device's size be substantial?

Additional discussion was added on this topic. The current device was designed with small animals in mind (rats, but they can be upscaled to accommodate clinically relevant dimensions and IEQs).

To achieve insulin independence in man islet isolation center typically transplant around 5000 IEQ/kg for an 80kg person this would mean 400.000 IEQ to reach insulin independence. Unfortunately, numerous donor and retrieval factors, such as donor age, body mass index (BMI), cold ischemia time, collagenase lot, organ perfusion quality, or retrieval team can influence the outcome of the islet isolation process and affect islet yields. Therefore, islet isolation centers typically use what they get for transplantation and 5000 IEQ/KG is considered an optimal number but can sometimes be lower or higher in practice. In light of clinical trials going on with devices in the field (viacyte’s open device for example), multiple smaller devices are probably a more likely strategy used in the future. This builds in additional safety if one of many fails the others can still function. One large device for 400.000 IEQ is not considered a likely strategy in the field.

Line 597 and Line 599 figure cited (Fig. 4L) refers to the area under the oxygen diffusion curve.

Corrections were made accordingly

Lines 598-599 “On the other hand, PVDF/PVP membranes with 110 nm pores, similar to the MailPan® devices with 100 nm pores, allow the diffusion of IgG (Fig. 4L and 1H).39 Next, we compared the immune protection” Is there a contradiction with what was said above?

We rephrased this sentence since the reviewer is right it’s not a contradiction but a confirmation of similarity.

Line 425: “For IgG only 1.6 ± 0.7 % permeated through the PVDF/PVP membrane, but IgG could not be detected in the receiver chamber of the PVDF-alone membrane, suggesting no diffusion (Fig.4J)”

This sentence was rewritten to better clarify the differences between the two membranes

Line 604 Authors do not demonstrate the device's ability to block the circulation of pro-inflammatory chemokines and cytokines.

Please see changes we addressed above regarding this topic

Minor revisions:

Line 89-91 “Based on these findings, we selected polyvinylidene fluoride (PVDF) since it induces relatively low oxidative stress in beta-cells compared to the other tested biomaterial….” Add references.

Lines 106-107 “We showed that primary human islets remained viable and functional for 8 days, like free-floating controls.” Have other times been evaluated ?

Line 320 and Line 481 and FIG7C;7D In addition to the stimulation index, values of insulin concentration per microgram of total DNA or protein should also be reported.

Page 19 Figure 1 G,H,I It is preferable to use the same units on the ordinates for clarity.

Page 24 Figures 4 B) 4 E) check the scale in the ordinate since it does not start from zero as with the other graphs.

We thank the reviewer for a thorough check on these topics. Similar to some of the comments mentioned above we have now addressed and improved the above issues
