# Supplementary material for: Supporting islet function in a PVDF membrane based macroencapsulation delivery device by solvent non-solvent casting using PVP
Source: PLoS One. 2025 Mar 12;20(3):e0298114. doi: 10.1371/journal.pone.0298114 (PMC11902058; doi:10.1371/journal.pone.0298114)
Supplement: Attachment — Submitted filename: answers to reviewers.docx [file pone.0298114.s007.docx]

The manuscript titled: ‘’Supporting islet function in a PVDF membrane based immunoprotective cell delivery device by solvent non-solvent casting using PVP’’ by Denise F.A. de Bont et al., focuses on the optimization of membrane fabrication PVDF-PVP ratio with respect to the diffusion properties of the device that can be tailored to support human islet function while preventing at the same time the membrane entry of primary human macrophages that damaged beta cells.

Even if the study presented the findings of original research focusing on new therapeutic approaches for Type 1 diabetic (T1D) patients who are lifelong dependent on insulin therapy to keep their blood glucose levels under control, the experiments, particularly the in vitro experiment, and other analyses were not performed to a high technical standard and were not presented and described in sufficient detail. Consequently, the conclusions are not supported by the appropriate data. A large quantity of data must be added to support the authors' hypothesis, which now is missing from the text.

Major points:

-There is a need of a test that validates the presence of pure islets after culture on the device (e.g., the Dithizone assay), as well as an evaluation of their functioning throughout time in culture and morphologic characterization. I suggest the gene expression characterization for the islets that are cultured in PVDF-PVP device at short and long time in culture.

-In the manuscript are used INS-1E rat insulinoma cells, machophages and human islets. While the function of using macrophages is obvious, it is not clear why, in the last paragraph, human islet cells are used in place of the INS-1E rat insulinoma cells that were previously studied for cytotoxicity of membranes. In the paragraph and the discussion section, I suggest a more thorough explanation of the experimental strategy.

-The goal of the manuscript is preventing the entry of primary human macrophages that damage beta cells with the use of this device, but in the paragraph ‘’diffusion of small molecules through membranes’’ the authors demonstrated the capacity of IgG to pass through the membrane. This event could damage the efficacy of the human islet functionality? I suggest discussing this point and include new experiments that verified this point.

-Moreover, I suggest reviewing the material and method section. Experiments are not described in an appropriate manner. in particular in the MTT assay paragraph, what do you mean for extraction samples? Are they the device with the different cells or the cells are removed from the device and then plated alone on the 96 well in order to obtain 50% of the confluent monolayer?

Consider explaining the experiments more carefully.

Minor points:

-In the manuscript are present some mistakes (e.g. line 172). I request a spell-check of the text.
